# Ginsenoside Rb1 for Myocardial Ischemia/Reperfusion Injury: Preclinical Evidence and Possible Mechanisms

**DOI:** 10.1155/2017/6313625

**Published:** 2017-12-21

**Authors:** Qun Zheng, Xiao-Yi Bao, Peng-Chong Zhu, Qiang Tong, Guo-Qing Zheng, Yan Wang

**Affiliations:** Department of Cardiology, The Second Affiliated Hospital and Yuying Children's Hospital of Wenzhou Medical University, Wenzhou, China

## Abstract

Ginseng is an important herbal drug that has been used worldwide for many years. Ginsenoside Rb1 (G-Rb1), the major pharmacological extract from ginseng, possesses a variety of biological activities in the cardiovascular systems. Here, we conducted a preclinical systematic review to investigate the efficacy of G-Rb1 for animal models of myocardial ischemia/reperfusion injury and its possible mechanisms. Ten studies involving 211 animals were identified by searching 6 databases from inception to May 2017. The methodological quality was assessed by using the CAMARADES 10-item checklist. All the data were analyzed using RevMan 5.3 software. As a result, the score of study quality ranged from 3 to 7 points. Meta-analyses showed that G-Rb1 can significantly decrease the myocardial infarct size and cardiac enzymes (including lactate dehydrogenase, creatine kinase, and creatine kinase-MB) when compared with control group (*P* < 0.01). Significant decrease in cardiac troponin T and improvement in the degree of ST-segment depression were reported in one study (*P* < 0.05). Additionally, the possible mechanisms of G-Rb1 for myocardial infarction are antioxidant, anti-inflammatory, antiapoptosis, promoting angiogenesis and improving the circulation. Thus, G-Rb1 is a potential cardioprotective candidate for further clinical trials of myocardial infarction.

## 1. Introduction

Myocardial infarction (MI) is the most severe manifestation of coronary artery disease (CAD), which can pathologically cause cardiomyocyte death due to prolonged ischemia. CAD accounts for over seven million deaths globally per year [1]. The economic impact of MI is substantial. The direct cost of hospitalization in the United States is at least $450 billion and the loss of productive life years is also tremendous [2]. Prompt restoration of blood flow is crucial to salvage ischemic myocardium [3]. Reperfusion strategies such as primary percutaneous coronary intervention (PCI) and thrombolysis have shown to reduce mortality and infarct size and improve left ventricular function; however, reperfusion itself may result in adverse events [4]. Abrupt restoration therapy of coronary flow can lead to reversible impairment of myocardial contractility (myocardial stunning), ventricular arrhythmias, and microvascular dysfunction. The pattern of injury that is inflicted on the myocardium has been termed as myocardial ischemia/reperfusion (I/R) injury [5], and the accumulating deleterious effects lead to myocyte necrosis and impaired cardiomyocyte healing, eventually contributing to heart failure associated with other poor outcomes [6]. Consequently, it remains a priority to seek new cardioprotective strategies to improve myocardial salvage and cardiac function.

**Figure 1:**
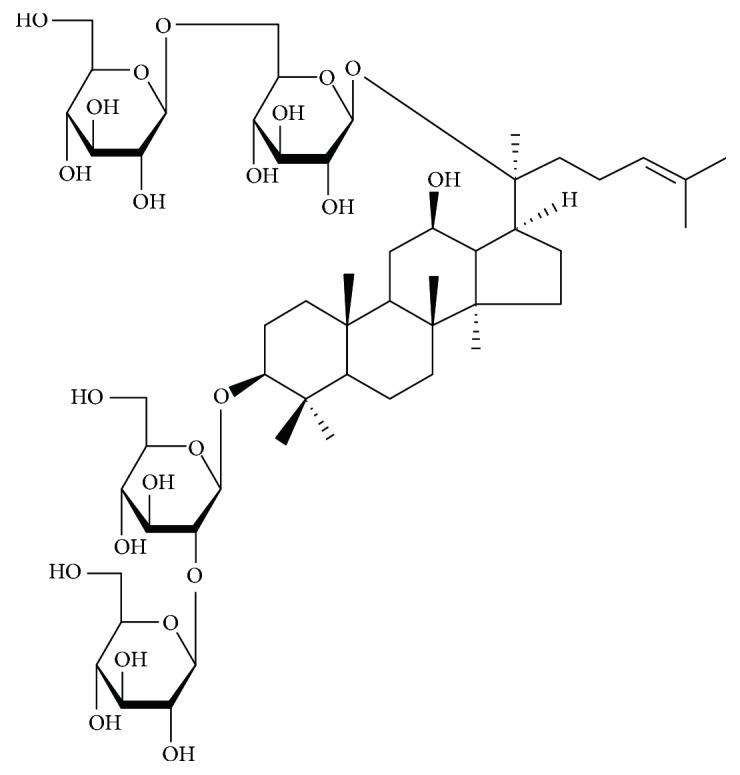
Chemical structures of ginsenoside Rb1.

Ginseng as an important herbal drug has been worldwide used in oriental countries for thousands of years and is also one of the most extensively botanical products used in other areas in the world [7]. Ginsenosides, the triterpene saponins, is one of the major components of ginseng [8]. To date, more than 30 kinds of ginsenosides have been identified [9]. Ginsenoside Rb1 (G-Rb1) (the major pharmacological extract and chemical structure is shown in Figure 1) possesses a variety of biological activities in the cardiovascular systems probably through antioxidant, anti-inflammatory, and antiapoptosis [10], promoting angiogenesis [11] and an antiarrhythmic effect [12], suppressing ventricular remodeling after acute MI [13], and inhibiting hypertrophy and ventricular hypertrophy [14]. However, the efficacy and mechanisms of G-Rb1 for experimental MI have not been systematically evaluated yet. In addition, systematic review of animal data can provide preclinical evidence for the potential translational value from animal models to human disease [15]. Thus, the present study aims to evaluate the efficacy and mechanisms of ginsenosides Rb1 through experimental animal models with myocardial I/R injury.

## 2. Methods

### 2.1. Search Strategies

We searched EMBASE, PubMed, Cochrane Library, Wangfang database, China National Knowledge Infrastructure (CNKI), VIP database (VIP), and China Biology Medicine disc (CBM) from inception to May 2017. The following keywords were used: “Ginseng (MeSH Terms) OR Ginseng (Title/Abstract)” AND “myocardial infarction OR myocardial ischemia OR myocardial I/R OR myocardial I/R injury”. Moreover, reference lists of potential articles were searched for relevant studies. All the studies included were limited on animals.

### 2.2. Eligibility Criteria

The inclusion criteria were prespecified as follows: (1) G-Rb1 for animal models of myocardial I/R injury was established by ligating of the left anterior descending (LAD) coronary artery or injecting vasoconstrictor intravenously; (2) the treatment group received G-Rb1 as monotherapy at any dose, and interventions for control group were isasteric and nonfunctional liquid (normal saline) or no treatment; and (3) the primary outcome measures were MI size and/or cardiac enzymes and/or cardiac troponin T (cTnT) and/or the level of ST-segment depression and/or left ventricular ejection fraction (LVEF). The secondary outcome measures were mechanisms of G-Rb1 for myocardial I/R injury. The exclusion criteria were prespecified as follows: (1) not myocardial I/R model; (2) Combined use of other drugs; (3) no control group; and (4) duplicate publication.

### 2.3. Data Extraction

Two independent authors extracted the following details from the included studies: (1) the name of the first author and publication year; (2) the specific information of animals for each study, including animal species, number, sex, and weight; (3) model of myocardial I/R and the anesthesia methods for model preparation; (4) the information of treatment group, including therapeutic drug dosage, method of administration, duration of treatment, and the same information of control group; and (5) mean value and standard deviation of outcomes. The data of the highest dose was selected when the treatment group included various doses of the target drug. The result of the peak time point was included when the data were expressed at different times. Because some records' published data were only in graphical format, we made efforts to contact authors for further information. When response was not received, the numerical values were measured from the graphs by digital ruler software.

### 2.4. Risk of Bias in Individual Studies

We assessed the risk of bias by applying the ten-item scale [16] with minor modifications [17] as follows: (A) peer-reviewed publication; (B) control of temperature; (C) random allocation to treatment or control; (D) blinded induction of model; (E) blinded assessment of outcome; (F) use of anesthetic without significant intrinsic cardioprotective activity; (G) appropriate animal model (aged, diabetic, or hypertensive); (H) sample size calculation; (I) compliance with animal welfare regulations; and (J) statement of potential conflict of interests. Every item was given one point. Two authors independently evaluated the study quality, and divergences were well settled through consulting with correspondence authors.

### 2.5. Statistical Analysis

Heterogeneity across the subgroups was assessed using the Cochrane Q-statistic test (*P* < 0.05 was considered statistically significant) and the *I*^2^-statistic test. A fixed effects model (*I*^2^ < 50%) or a random effects model (*I*^2^ > 50%) was used depending on the value of *I*^2^. Funnel plots were used to visually estimate publication bias. We calculated the standard mean difference (SMD) with 95% confidence intervals (CIs). Sensitivity analyses omitting one study at a time from the original analysis were conducted to demonstrate our main results to be robust. The pooled analyses were carried out with RevMan 5.3 software. Ethical approval was not required for this type of literature research.

## 3. Results

### 3.1. Study Selection

**Figure 2:**
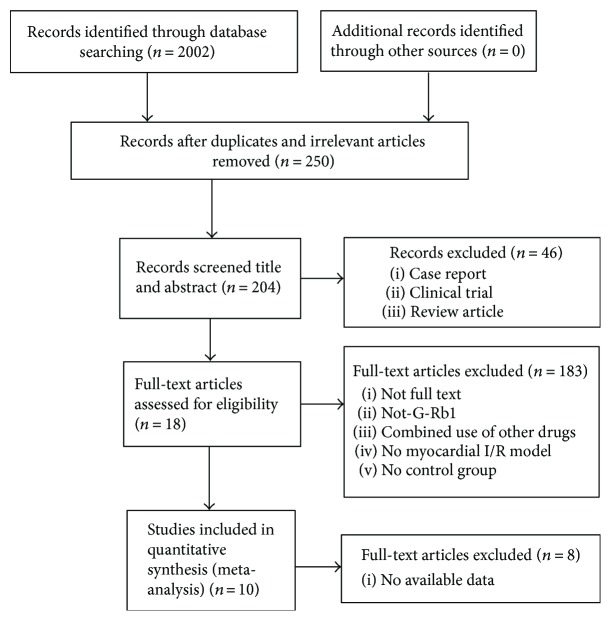
Summary of the process for identifying candidate studies.

A total of 2002 articles were retrieved through the pertinent literature retrieval from the database, of which 1752 were reduplicated and irrelevant articles. After screening titles and abstracts, 46 were excluded because they were (1) clinical trials; (2) case reports; or (3) review articles. We then studied the remaining 204 full-text articles. Among them, 186 articles were excluded because of at least one of the following reasons: (1) not a full text; (2) not G-Rb1; (3) no available data; (4) compared with traditional Chinese medicine; (5) no myocardial I/R model; or (6) no control group. Finally, 10 studies [18–27] were selected (Figure 2).

### 3.2. Characteristics of Included Studies

Eight studies [18–25] were published in Chinese, and 2 studies [26, 27] were published in English between 2003 and 2016. The 10 included studies involved adult Sprague-Dawley rats [18–24, 26, 27], and Wistar rats [25]. The weight of adult rats varied between 180 g and 320 g. Seven studies [18–20, 22, 24, 26, 27] used male animals and only three studies [21, 23, 25] used both female and male rats. To induce anesthesia, 2 studies [21, 24] used chloral hydrate; 3 studies [18, 23, 26] used pentobarbital sodium; 2 studies [22, 27] used barbital sodium; 2 studies [20, 25] used ethyl ether; and 1 study [19] used urethane. Nine myocardial I/R models were produced by ligation of the LAD; and the remaining one [21] was produced by intravenous isoprenaline (30 mg·kg^−1^). All studies implemented the dose gradient of Ginseng. Among them, 5 studies [18, 19, 22, 26, 27] utilized 40 mg·kg^−1^; 2 studies [21, 23] adopted 20 mg·kg^−1^; 1 study [24] used 4 mg·kg^−1^·d^−1^; and the remaining 1 study [20] used 100 mg·kg^−1^. Eight studies [18–20, 22–24, 26, 27] utilized MI size as outcome measure, and myocardial cell apoptosis rate in 2 studies [22, 27]. The level of ST-segment depression was reported in 1 study [21], but LVEF was not mentioned. Lactate dehydrogenase (LDH) was reported in 4 studies [18, 21, 26, 27], creatine kinase (CK) in 3 studies [18, 21, 26], creatine kinase-MB (CK-MB) in 2 studies [26, 27], cTnT in 1 study [26], superoxide dismutase (SOD) in 4 studies [18, 20, 21, 24], malondialdehyde (MDA) in 4 studies [18, 20, 21, 24], phosphothreonine kinase (p-Akt) in 3 studies [22, 26, 27], caspase-3 in 2 studies [19, 27], vascular endothelium growth factor (VEGF) in 1 study [23], NO in 2 studies [20, 21], glutathione (GSH) in 1 study [21], and tumor necrosis factor-*α* (TNF-α) in 1 study [19]. The overall characteristics of included publications are shown in Table 1.

### 3.3. Study Quality

The score of study quality ranged from three to seven in a total of ten points. Of which, 6 studies [19–21, 23–25] got three points; 2 studies [18, 22] got four points; 1 study [26] got six points; and 1 study [27] got seven points. All the included records were peer-reviewed publications, and all animals were allocated randomly to treatment group and control group; however, no study reported a sample size calculation, blinded induction of model, and blinding their assessment of outcome. Three included studies [18, 22, 27] were performed on diabetic rats and others on healthy rats. Control of temperature was described in 2 studies [26, 27]. All studies used an anesthetic without intrinsic cardioprotective properties; 2 studies [26, 27] reported compliance with animal welfare regulations, and 2 studies [26, 27] declared no potential conflict of interests. The methodological quality is concluded in Table 2.

### 3.4. Effectiveness

#### 3.4.1. MI Size

**Figure 3:**
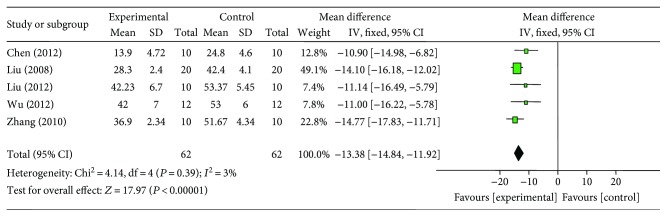
The forest plot: effects of ginsenoside Rb1 for decreasing the myocardial infarction size compared with control group.

Meta-analysis of 8 studies [18–20, 22–24, 26, 27] showed significant effect of G-Rb1 for decreasing the MI size compared with control group (*n* = 85, MD −12.45, 95% CI: −15.87 to −9.02, *P* < 0.00001; heterogeneity: *χ*^2^ = 53.71, df = 7 (*P* < 0.00001), *I*^2^ = 87%). Owing to obvious heterogeneity, we used sensitivity analyses and removed the respective outlier study. After removing two studies [19, 24] that do not utilize infarct area/area at risk (IA/AR) to calculate MI size and 1 study [26] that MI experimental model was induced by blocking LAD for 45 minutes compared to 30 minutes in other studies, meta-analysis of 5 studies [18, 20, 22, 23, 27] showed significant effect of G-Rb1 for decreasing the MI size compared with control group (*n* = 56, MD −13.38, 95% CI: −14.84 to −11.92, *P* < 0.00001; heterogeneity: *χ*^2^ = 4.14, df = 4 (*P* = 0.39), *I*^2^ = 3%) (Figure 3).

#### 3.4.2. Cardiac Enzymes and/or cTnT

**Figure 4:**
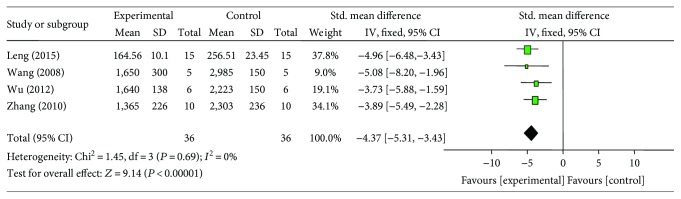
The forest plot: effects of ginsenoside Rb1 for decreasing lactate dehydrogenase compared with control group.

**Figure 5:**
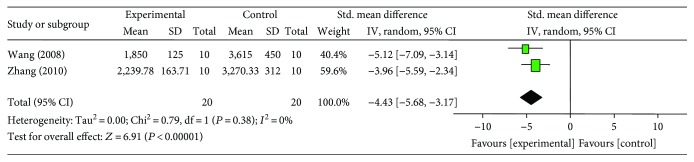
The forest plot: effects of ginsenoside Rb1 for decreasing creatine kinase compared with control group.

Meta-analysis of 4 studies [18, 21, 26, 27] showed significant effect of G-Rb1 for decreasing the LDH compared with control group (*n* = 36, SMD −4.37, 95% CI: −5.31 to −3.43, *P* < 0.00001; heterogeneity: *χ*^2^ = 1.45, df = 3 (*P* = 0.69), *I*^2^ = 0%) (Figure 4). Meta-analysis of 3 studies [18, 21, 26] showed a significant effect of G-Rb1 for decreasing CK compared with control group (*n* = 29, MD −3.62, 95% CI: −5.35 to −1.90, *P* < 0.00001; heterogeneity: *χ*^2^ = 7.05, df = 2 (*P* < 0.00001), *I*^2^ = 72%). After sensitivity analyses, we removed 1 study [21] that utilized both male and female rats. Meta-analysis of 2 studies [18, 26] showed that G-Rb1 had significant effects for reducing CK compared with control group (*n* = 20, SMD −4.43, 95% CI: −5.68 to −3.17, *P* < 0.00001; heterogeneity: *χ*^2^ = 0.79, df = 1 (*P* = 0.38), *I*^2^ = 0%) (Figure 5). G-Rb1 in two studies [26, 27] showed the significant effects for reducing CK-MB compared with control (*P* < 0.05 or *P* < 0.01) according to CK-MB as outcome measures; however, they failed to pool analysis because 1 study [27] was set up in diabetic rat model and myocardial I/R model was induced by blocking LAD for 45 minutes compared to 30 minutes in another study [26]. Only 1 study [26] showed that G-Rb1 had significant effects to reduce cTnT compared with control group (*P* < 0.05).

#### 3.4.3. The Level of ST-Segment Depression and LVEF

One study [21] reported that G-Rb1 can improve the ST-segment depression compared with control (*P* < 0.05) according to the change of ST segment in electrocardiogram as an outcome measure. There was no study involving LVEF as outcome measure.

### 3.5. Subgroup Analysis

**Figure 6:**
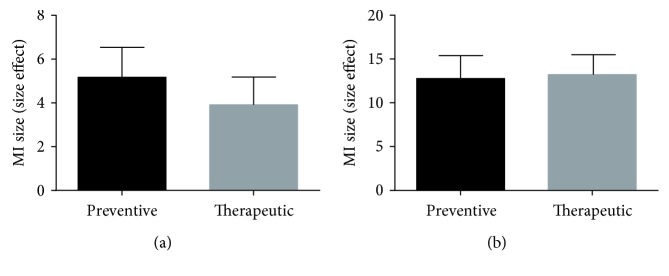
(a) Subgroup analysis of myocardial infarction size by preventive and therapeutic dose (*P* > 0.05). (b) Subgroup analysis of lactate dehydrogenase by preventive and therapeutic dose (*P* > 0.05).

**Figure 7:**
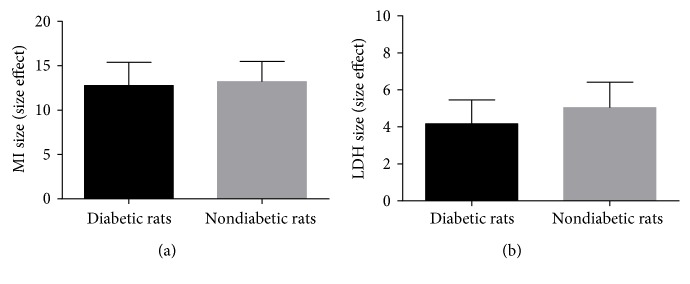
(a) Subgroup analysis of myocardial infarction size by diabetic rats and nondiabetic rats (*P* > 0.05). (b) Subgroup analysis of lactate dehydrogenase by diabetic rats and nondiabetic rats (*P* > 0.05).

Five studies [18, 19, 22, 26, 27] reported that G-Rb1 was given before establishing model, and five other studies [20, 21, 23–25] reported it was given after establishing model. We conducted subgroup analysis, and the result of the meta-analysis (Figure 6) showed that there was no difference in MI size effect size and LDH effect size between preventive and therapeutic effects of G-Rb1. In consideration of different administration time, which may lead to an inaccurate assessment of the effects of the intervention [28], thus we suggest that both preventive and therapeutic effects of G-Rb1 for myocardial I/R injury should be performed further in the animal models. Three studies [18, 22, 27] reported the effects of G-Rb1 in MI as a result of diabetes. We conducted a subgroup analysis, and the result of meta-analysis (Figure 7) showed that there was no difference in MI size effect size and LDH effect size between diabetic rats and nondiabetic rats.

### 3.6. Cardioprotective Mechanisms

**Figure 8:**
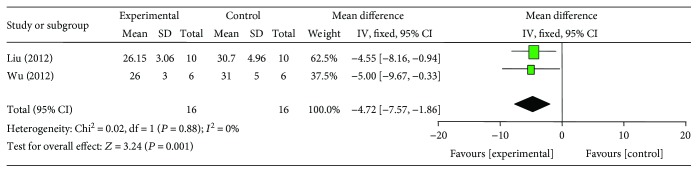
The forest plot: effects of ginsenoside Rb1 for decreasing myocardial cell apoptosis rate compared with control group.

**Figure 9:**
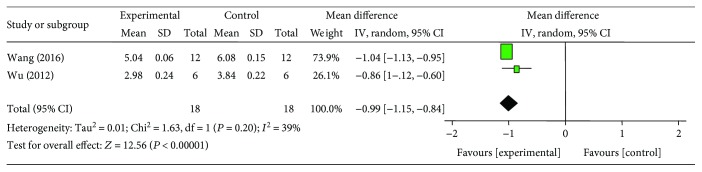
The forest plot: effects of ginsenoside Rb1 for decreasing caspase-3 compared with control group.

**Figure 10:**
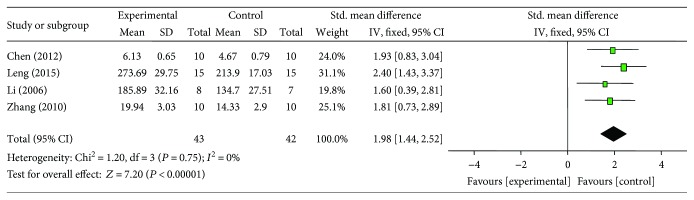
The forest plot: effects of ginsenoside Rb1 for increasing superoxide dismutase compared with control group.

**Figure 11:**
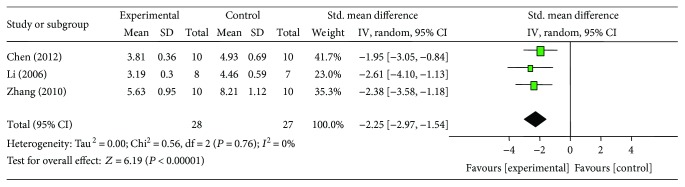
The forest plot: effects of ginsenoside Rb1 for decreasing malondialdehyde compared with control group.

**Figure 12:**
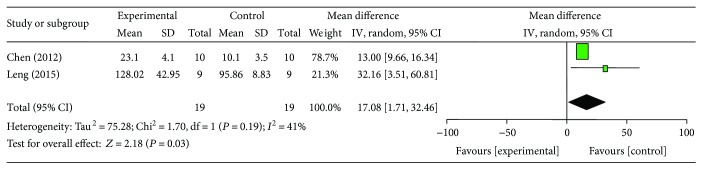
The forest plot: effects of ginsenoside Rb1 for increasing NO compared with control group.

**Figure 13:**
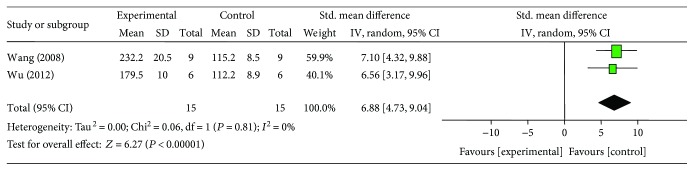
The forest plot: effects of ginsenoside Rb1 for increasing phosphothreonine kinase compared with control group.

**Figure 14:**
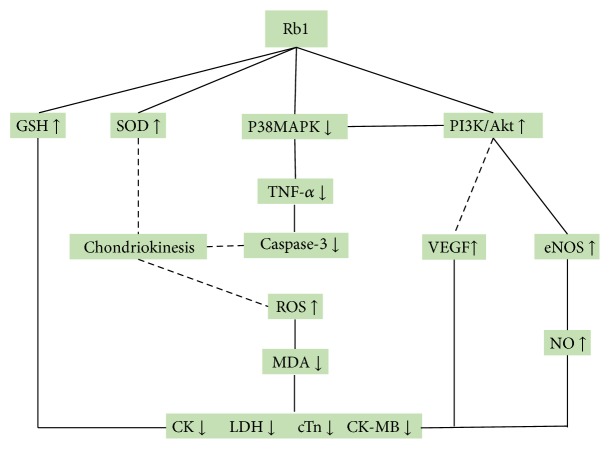
A schematic representation of cardioprotective mechanisms of ginsenoside Rb1 for myocardial ischemia/reperfusion injury. Solid lines indicate established effects, whereas dashed lines represent putative mechanisms.

Compared with controls, meta-analysis of 2 studies [22, 27] showed that G-Rb1 significantly decreased myocardial cell apoptosis rate (*n* = 16, MD −4.72, 95% CI: −7.57 to −1.86, *P* < 0.00001; heterogeneity: *χ*^2^ = 0.02, df = 1 (*P* = 0.88), *I*^2^ = 0%) (Figure 8); 2 studies [19, 27] for reducing caspase-3 (*n* = 18, SMD −1.02, 95% CI: −1.11 to −0.93, *P* < 0.00001; heterogeneity: *χ*^2^ = 1.63, df = 1 (*P* = 0.20), *I*^2^ = 39%) (Figure 9); 4 studies [18, 20, 21, 24] for increasing SOD (*n* = 42, SMD 1.98, 95% CI: 1.44 to 2.52, *P* < 0.00001; heterogeneity: *χ*^2^ = 1.20, df = 3 (*P* = 0.75), *I*^2^ = 0%) (Figure 10); 3 studies [18, 20, 24] for reducing MDA (*n* = 28, SMD −2.25, 95% CI: −2.97 to −1.54, *P* < 0.00001; heterogeneity: *χ*^2^ = 0.56, df = 3 (*P* = 0.76), *I*^2^ = 0%) (Figure 11); 2 studies [20, 21] for increasing NO (*n* = 19, SMD 17.08, 95% CI: 1.71 to 32.46, *P* < 0.00001; heterogeneity: *χ*^2^ = 1.70, df = 1 (*P* = 0.19), *I*^2^ = 41%) (Figure 12); 2 studies [26, 27] for increasing p-Akt (*n* = 15, SMD 6.88, 95% CI: 4.73 to 9.04, *P* < 0.00001; heterogeneity: *χ*^2^ = 0.06, df = 1 (*P* = 0.81), *I*^2^ = 0%) (Figure 13); 1 study [19] for reducing TNF-*α* (*P* < 0.05); 1 study [21] for increasing GSH (*P* < 0.05); and 1 study [23] for increasing VEGF (*P* < 0.05). We summarized a schematic representation of cardioprotective mechanism of G-Rb1 for myocardial I/R injury (Figure 14).

## 4. Discussion

### 4.1. Summary of Evidence

This is the first preclinical systematic review to estimate the efficacy of G-Rb1 for myocardial I/R injury. Ten studies with 221 animals were selected. The quality of studies included were generally moderate. The evidence available from present study showed that G-Rb1 exerted potential cardioprotective function in acute MI largely through antioxidant, anti-inflammatory, anti-apoptosis, promoting angiogenesis, and improvement of the circulation. Despite the apparent positive results, we should interpret them with caution because of the methodological flaws.

### 4.2. Limitations

First, we only searched in English and Chinese studies, which may lead to a certain degree of selective bias. Second, negative findings were less likely to be published. Thus, the dominance of positive studies might lead to the efficacy being overestimated. Third, study quality was considered as moderate, which ranged from 3 to 7 points, indicating that the results should be explained with caution. In addition, MI generally occurs in patients with medical problems, such as old age, diabetes, hypertension, and hyperlipidemia [29]. However, only 3 studies [18, 22, 27] structured a myocardial I/R model in diabetic rats. Finally, another weakness is the heart protection of estrogen. Three included studies [21, 23, 25] adopted female animals. Although the mechanism is unclear, the heart protective effect of estrogen has been reported both in clinical and preclinical studies [30].

### 4.3. Implications

Ideal coronary artery thrombosis model should include the following points: (1) provide stable thrombus; (2) combined with intimal injury; (3) low mortality; (4) suitable for thrombolytic therapy; (5) and simple to operate [31]. Up to now, there has been several frequently used methods to establish animal models of MI: (1) coronary artery stenosis or occlusion is directly caused by ligation of coronary artery. After coronary artery ligation, the ECG and pathological findings were corresponding, most of which were extensive anterior wall MI and showed good reproducibility and stability [32]. Currently, it is the most recognized and commonly used model of MI; however, establishment of this animal model needs high proficiency; (2) coronary artery stenosis or occlusion is caused by intravenous injection of vasoconstrictor drugs [33]. The establishing procedure of this method is simple, mainly diffuse myocardial injury in the whole heart, rather than the local myocardial injury. Obviously different from physiological process of MI, resulting in that it is difficult to position, and quantitative observation for later evaluation of MI area [34]; (3) stimulate coronary artery adventitia to induce thrombus by controlled micro current stimulator [35]. This model is seminal for structural models, causing a better understanding of vascular biomechanics. However, the occlusion is permanent; (4) coronary artery stenosis and infarction are caused by coronary artery atherosclerosis. It is most consistent with human physiology. The weaknesses are long test cycle and high mortality, and thus this method has been used rarely [36]. In this review, nine studies [18–20, 22–27] reported coronary artery stenosis or occlusion by ligation of coronary artery except for one study [21] by intravenous injection of isoprenaline, indicating that the uniform animal model was rational for improving the reliability of the experimental results.

The choice of animal depends on the purpose of the experiment. Factors that need to be considered include technical requirements, availability of the animal, cost, and ethical considerations [37]. The commonly used animal models in MI experiments are listed as follows: rat, mouse, rabbit, pig, dog, cat, monkey, and so forth. Rodents like rat and mouse have the advantages of being cheap and easy to anaesthetize and work with a variety of strains with specific atherosclerotic risk factors such as hypertension, and ECG, serum enzymes, and other indicators can be measured [32]. However, when it comes to performing surgery, their small size presents quite a technical challenge. The main area in which this animal model has been used is in the development of cardiac tissue and in understanding basic molecular mechanisms that control normal heart development and subsequent congenital cardiovascular malformations [38]. Rabbits have the convenience of reasonable anatomical size and lower cost in terms of purchase and maintenance. However, they are also not as easy to operate compared to the large animals because of the smaller size of the vasculature [37]. The dog and cat continue to be a mainstay of cardiovascular research. The reasons include a convenient size of between 10 and 30 kg, a ready supply, and the fact they are easy to work with. Again, their lack of rapid growth means they are useful for long-term *in vivo* studies. However, obtaining licenses for the use of dogs and cats has become difficult [37]. Pigs are popular for experimental study, as they have similar anatomy and physiology to that of humans [39]. They can develop spontaneous atherosclerosis [40]. Nevertheless, they are capable of rapid growth. A typical example is that of a 25 kg pig which after 8 weeks will grow to around 100 kg [37]. From both an anatomical and physiological standpoint, primates like monkey resemble humans more closely than any other type of animals. Due to the lack of dramatic growth, they are ideal for very long-term studies, usually over several years. However, ethical considerations restrict their use in experimentation [41]. Furthermore, they are expensive to purchase and to maintain when compared with other animals species [37]. In this study, all studies utilized rat to establish animal model. It was considered as a good choice for small- and medium-sized research institute to test investigate the efficacy of G-Rb1 for animal models of myocardial I/R injury and its possible mechanisms. Thus, we suggest further that the animal models for MI should be chosen according to the experimental purpose and conditions.

Various pharmacological agents have been shown to reduce myocardial I/R injury in animal models [42]; however, lack of cardioprotectant has been routinely used for clinical myocardial I/R injury so far. Thus, translation of a cardioprotectant for myocardial I/R injury from the bench to the bed is urgently needed [42–44]. In the present study, the findings showed that G-Rb1 significantly decreased the MI size and cardiac enzymes, decreased cardiac cTnT, and increased the decline degree in ST-segment. Therefore, the present study provides a preclinical evidence-based approach to develop G-Rb1 for acute MI. Cardioprotection by anti-inflammation, antioxidant, antiapoptosis, and improving the circulation for myocardial I/R injury [45] was an innovative strategy for antagonizing the injurious biochemical and molecular events that eventually resulted in irreversible ischemic injury [46]. In the present review, the possible mechanisms of G-Rb1 for myocardial I/R injury are summarized as follows: (1) inhibition of apoptosis through downregulating the expression of caspase-3 [19, 27] via inhibiting p38MAPK signaling pathway [47]; (2) antioxidant through increasing GSH [21] and enhancing SOD-induced antioxidant via attenuating chondriokinesis to reduce the release of MDA [18, 20, 21, 24]; (3) anti-inflammatory through inhibiting the expression of TNF-*α* [19] by inhibiting the activation of PI3K/Akt signaling, inhibiting p38MAPK and crosstalk between the two signaling pathways [35]; (4) promotion of angiogenesis through enhancing VEGF [23]; and (5) improvement of the circulation by increasing the expression of NO via the activation of PI3K/Akt signaling [20, 21]. Thus, we summarized a schematic representation of cardioprotective mechanism of G-Rb1 for myocardial I/R injury through anti-inflammation, antioxidant, antiapoptosis, promoting angiogenesis, and improving the circulation.

However, there are increasing concerns that poor experimental design, and transparent reporting contribute to the frequent failure of translating preclinical discoveries into novel treatments for human disease [48]. In the present study, the quality of including studies was considered as moderate, which ranged from three to seven in a total of ten points. The main drop points are that no study reported a sample size calculation, blinded induction of model, and blinding their assessment of outcome. And only three included studies were performed on diabetic rats, in which the conditions of clinical MI patients with morbidity may not accurately be replicated owning to MI generally occurring in elders with comorbidities such as diabetes, hyperglycemia, or hypertension [29]. Reporting guidelines set a checklist format of predetermined criteria for more complete and transparent reports of biomedical research, and thus increasing their value to inform policy, scientific practice, and clinical practice [49]. The animal research: “Reporting *in vivo* experiments (ARRIVE) guidelines” [50] provides guidance on complete and transparent reporting of *in vivo* animal research, which aims to improve the quality of research reports. Up to 2014, the ARRIVE guidelines have been endorsed by over 300 research journals around the world, including the Nature Publishing Group, BioMed Central, and PLOS [51]. Thus, we suggest that further design and reporting of the studies should refer to the ARRIVE guidelines, and sample size calculation, blinded induction of model, and blinding their assessment of outcome should be focused on [16]. Animal research should be designed with a clinical perspective in mind, including both genders, study a broad age-range, and document the effect of clinically prevalent comorbidity and comedication on effect size. Animal research should be registered prior to its execution in a generally accessible database similar to human (drug) research (http://www.clinicaltrials.com). This would help to provide a more informed view before proceeding to clinical trials and reduce publication bias [52]. It allows verification of predefined study hypothesis and end-points of the study [53].

It is well known that animal experiments have contributed to our understanding of mechanisms of diseases, but the translation of preclinical experiment which results in a prediction of the effectiveness of treatment strategies in clinical trials is still challenging [48, 52]. High-quality RCTs are commonly regarded the gold standard in judging the treatment efficacy and safety of interventions [54]. Given the huge gap between the animal studies and the clinical trials, the rigorous RCTs of G-Rb1 are needed.

## 5. Conclusion

The findings of the present study demonstrated that G-Rb1 exerted cardioprotective function in acute MI largely through antioxidant, anti-inflammatory, and antiapoptosis, promoting angiogenesis and improving the circulation. Thus, G-Rb1 is a potential cardioprotective candidate for further clinical trials of MI.

## Figures and Tables

**Table 1:** Characteristics of the 10 included studies.

Study (year)	Species (sex, *n* = experimental/control group)	Weight	Model (method)	Anesthetic	Treatment group (method to astragal sides)	Control group	Outcome index (time)	Intergroup differences
Zhang (2010)	SD rats (male, 10/10)	220–280 g	Block LAD for 30 minutes then reflow for 120 minutes	Pentobarbital sodium (50 mg/kg, 3%)	Intravenous injection G-Rb1 (40 mg/kg) 10 minutes earlier before reperfusion	Intravenous injection isasteric and nonfunctional liquid 10 minutes earlier before reperfusion	(1) Myocardial infarct size;	(1) *P* < 0.05
							(2) LDH	(2) *P* < 0.05
							(3) CK	(3) *P* < 0.05
							(4) MDA	(4) *P* < 0.05
							(5) SOD	(5) *P* < 0.05
							(6) HR	(6) *P* < 0.05
							(7) RPP	(7) *P* < 0.05
							(8) MAP	(8) *P* < 0.05

Wang (2016)	SD rats (male, 12/12)	180–240 g	Block LAD for 30 minutes then reflow for 120 minutes	Urethane (1 ml/kg, 10%)	Intravenous injection G-Rb1 (40 mg/kg) before reperfusion	Intravenous injection nothing before reperfusion	(1) Myocardial infarct size	(1) *P* < 0.05
							(2) Caspase-3	(2) *P* < 0.05
							(3) TNF-*α*	(3) *P* < 0.05

Chen (2012)	SD rats (male, 10/10)	220–260 g	Block LAD for 30 minutes then reflow for 180 minutes	Ethyl ether	Intravenous injection G-Rb1 (100 mg/kg·d), once a day, for 7 days, before establishing model	Intravenous injection isasteric normal saline, once a day, for 7 days, before establishing model	(1) Myocardial infarct size	(1) *P* < 0.01
							(2) SOD	(2) *P* < 0.01
							(3) MDA	(3) *P* < 0.01
							(4) NO	(4) *P* < 0.05

Leng (2015)	SD rats (male/female, 15/15)	180–220 g	Intravenous injection isoprenaline (30 mg/kg)	Chloral hydrate (10%)	Intravenous injection G-Rb1 (20 mg/kg·d), once a day, for 5 days, before establishing model	Intravenous injection isasteric normal saline, once a day, for 5 days, before establishing model	(1) Microcirculation blood flow of heart surface	(1) *P* < 0.01
							(2) LDH	(2) *P* < 0.01
							(3) CK	(3) *P* < 0.01
							(4) MDA	(4) *P* < 0.01
							(5) SOD	(5) *P* < 0.01
							(6) GSH	(6) *P* < 0.01
							(7) NO	(7) *P* < 0.01
							(8) HR	(8) *P* < 0.01
							(9) The level of ST-segment depression	(9) *P* < 0.01

Liu (2012)	SD rats (male, 10/10)	250–300 g	Block LAD for 30 minutes then reflow for 120 minutes	Barbital sodium (50 mg/kg)	Intravenous injection G-Rb1 (40 mg/kg) 10 minutes earlier before reperfusion	Intravenous injection isasteric normal saline 10 minutes earlier before reperfusion	(1) Myocardial infarct size	(1) *P* < 0.05
							(2) Myocardial cell apoptosis rate	(2) *P* < 0.05
							(3) P-Akt	(3) *P* < 0.05

Liu (2008)	SD rats (male/female, 20/20)	200–250 g	Block LAD for 30 minutes	Pentobarbital sodium (1%)	Intravenous injection G-Rb1 (20 mg/kg·d), once a day, for 7 days, before establishing model	Intravenous injection isasteric normal saline, once a day, for 7 days, before establishing model	(1) Myocardial infarct size	(1) *P* < 0.01
							(2) VEGF	(2) *P* < 0.01
							(3) Microvessel density	(3) *P* < 0.01
							(4) HR	(4) *P* < 0.01

Li (2006)	SD rats (male, 7/8)	200–250 g	Block LAD for 30 minutes	Chloral hydrate (300 mg/kg)	Intravenous injection G-Rb1 (4 mg/kg·d), once a day, for 7 days, before establishing model	Intravenous injection isasteric normal saline, once a day, for 7 days, before establishing model	(1) Myocardial infarct size	(1) *P* < 0.01
							(2) Left ventricular mass index	(2) *P* < 0.05
							(3) The left ventricle section diameter	(3) *P* < 0.01
							(4) SOD	(4) *P* < 0.01
							(5) MDA	(5) *P* < 0.05
							(6) HR	(6) *P* < 0.05

Zeng (2003)	Wistar rats (male/female, 5/5)	200–250 g	Block LAD for 30 minutes then reflow for 360 minutes	Ethyl ether	Intravenous injection G-Rb1 (20 mg/kg) 10 minutes earlier before establishing model	Intravenous injection isasteric normal saline 10 minutes earlier before establishing model	(1) Myocardial apoptosis cell count	(1) *P* < 0.01

Wang (2008)	SD rats (male, 9/9)	260–320 g	Block LAD for 45 minutes then reflow for 120 minutes	Pentobarbital sodium (80 mg/kg)	Intravenous G-Rb1 (40 mg/kg) 10 minutes earlier before reperfusion	Intravenous nothing before reperfusion	(1) Myocardial infarct size	(1) *P* < 0.01
							(2) CK	(2) *P* < 0.01
							(3) CK-MB	(3) *P* < 0.01
							(4) LDH	(4) *P* < 0.01
							(5) cTnT	(5) *P* < 0.01
							(6) HR	(6) *P* < 0.01
							(7) RPP	(7) *P* < 0.01
							(8) MAP	(8) *P* < 0.01

Wu (2012)	SD rats (male, 6/6)	250–300 g	Block LAD for 30 minutes then reflow for 120 minutes	Barbital sodium (50 mg/kg)	Intravenous G-Rb1 (40 mg/kg) 10 minutes earlier before reperfusion	Intravenous nothing before reperfusion	(1) Myocardial infarct size	(1) *P* < 0.05
							(2) Myocardial cell apoptosis rate	(2) *P* < 0.05
							(3) CK-MB	(3) *P* < 0.05
							(4) LDH	(4) *P* < 0.05
							(5) P-Akt	(5) *P* < 0.05
							(6) Caspase-3	(6) *P* < 0.05

SD rats: Sprague-Dawley; LAD: the left anterior descending coronary artery; G-Rb1: ginsenoside Rb1; SOD: superoxide dismutase; MDA: malondialdehyde; CK: creatine kinase; LDH: lactate dehydrogenase; CK-MB: creatine kinase-MB; cTnT: cardiac troponin T; HR: heart rate; RPP: rate-pressure product; MAP: mean arterial pressure; GSH: glutathione synthetase; VEGF: vascular endothelial growth factor.

**Table 2:** Risk of bias of the included studies.

Study	A	B	C	D	E	F	G	H	I	J	Total
Zhang (2010)	**√**		**√**			**√**	**√**				4
Wang (2016)	**√**		**√**			**√**					3
Chen (2012)	**√**		**√**			**√**					3
Leng (2015)	**√**		**√**			**√**				**√**	3
Liu (2012)	**√**		**√**			**√**	**√**				4
Liu (2008)	**√**		**√**			**√**					3
Li (2006)	**√**		**√**			**√**					3
Zeng (2003)	**√**		**√**			**√**					3
Wang (2008)	**√**	**√**	**√**			**√**			**√**	**√**	6
Wu (2012)	**√**	**√**	**√**			**√**	**√**		**√**	**√**	7

Studies fulfilling the criteria of the following: (A) peer reviewed publication; (B) control of temperature; (C) random allocation to treatment or control; (D) blinded induction of model; (E) blinded assessment of outcome; (F) use of anesthetic without significant intrinsic vascular protection activity; (G) appropriate animal model (aged, diabetic, or hypertensive); (H) sample size calculation; (I) compliance with animal welfare regulations; (J) statement of potential conflict of interests.

## References

[1] Mozaffarian D., Benjamin E. J., Go A. S. (2015). Heart disease and stroke statistics—2015 update: a report from the American Heart Association. *Circulation*.

[2] Weintraub W. S., Daniels S. R., Burke L. E. (2011). Value of primordial and primary prevention for cardiovascular disease: a policy statement from the American Heart Association. *Circulation*.

[3] Ribas N., Garcia-Garcia C., Merono O. (2017). Secondary prevention strategies after an acute ST-segment elevation myocardial infarction in the AMI code era: beyond myocardial mechanical reperfusion. *BMC Cardiovascular Disorders*.

[4] Heusch G., Gersh B. J. (2017). The pathophysiology of acute myocardial infarction and strategies of protection beyond reperfusion: a continual challenge. *European Heart Journal*.

[5] Seewald M., Coles J. J., Sigg D. C., Iaizzo P. A. (2017). Featured article: pharmacological postconditioning with delta opioid attenuates myocardial reperfusion injury in isolated porcine hearts. *Experimental Biology and Medicine*.

[6] Schonberger T., Jurgens T., Muller J. (2014). Pivotal role of phospholipase D1 in tumor necrosis factor-α-mediated inflammation and scar formation after myocardial ischemia and reperfusion in mice. *The American Journal of Pathology*.

[7] Guo Y. H., Zhao S., Du Y. X., Xing Q. J., Chen B. L., Yu C. Q. (2017). Effects of ginsenoside Rg1-loaded alginate-chitosan microspheres on human bone marrow stromal cells. *Bioscience Reports*.

[8] Palaniyandi S. A., Suh J. W., Yang S. H. (2017). Preparation of ginseng extract with enhanced levels of ginsenosides Rg1 and Rb1 using high hydrostatic pressure and polysaccharide hydrolases. *Pharmacogn Magazine*.

[9] Leung K. W., Cheung L. W., Pon Y. L. (2007). Ginsenoside Rb1 inhibits tube-like structure formation of endothelial cells by regulating pigment epithelium-derived factor through the oestrogen beta receptor. *British Journal of Pharmacology*.

[10] Lu J. M., Yao Q. Z., Chen C. Y. (2009). Ginseng compounds: an update on their molecular mechanisms and medical applications. *Current Vascular Pharmacology*.

[11] Kwok H. H., Chan L. S., Poon P. Y., Yue P. Y. K., Wong R. N. S. (2015). Ginsenoside-Rg1 induces angiogenesis by the inverse regulation of MET tyrosine kinase receptor expression through miR-23a. *Toxicology and Applied Pharmacology*.

[12] Li H., Xu J., Wang X., Yuan G. (2014). Protective effect of ginsenoside Rg1 on lidocaine-induced apoptosis. *Molecular medicine Reports*.

[13] Li C. Y., Deng W., Liao X. Q., Deng J., Zhang Y. K., Wang D. X. (2013). The effects and mechanism of ginsenoside Rg1 on myocardial remodeling in an animal model of chronic thromboembolic pulmonary hypertension. *European Journal of Medical Research*.

[14] Kong H. L., Li Z. Q., Yuan L. (2011). Ginsenosides Rb1 inhibits the hypertrophy of cardiomyocytes via nitric oxide synthase/nitric oxide system. *Guangdong Medical Journal*.

[15] Van J., Luijk M., Leenaars C. (2013). Towards evidence-based translational research: the pros and cons of conducting systematic reviews of animal studies. *ALTEX*.

[16] Macleod M. R., O’Collins T., Howells D. W., Donnan G. A. (2004). Pooling of animal experimental data reveals influence of study design and publication bias. *Stroke*.

[17] Yu L. J., Zhang K. J., Zhu J. Z. (2017). Salvianolic acid exerts cardioprotection through promoting angiogenesis in animal models of acute myocardial infarction: preclinical evidence. *Oxidative Medicine and Cellular Longevity*.

[18] Zhang L., Xia Z. Y. (2010). Protective effect of ginsenoside Rbl pre-conditioninlg on myocardium against ischemia-reperfusion injury in diabetic rats. *Chinese Journal of Medicinal Guide*.

[19] Wang Y., Zhang Y. Y. (2016). Protective effect and mechanisms of ginsenoside Rb1 on myocardial ischemia-reperfusion injury via p38MAPK signaling pathways. *World Latest Medicine Information*.

[20] Chen H. X. (2012). Protective effect of ginsenoside Rb1 on myocardial ischemia-reperfusion injury in rats. *Journal of Hubei University of Science and Technology*.

[21] Leng X., Zhang L. D., Jia L. Q. (2015). Effect of ginsenoside Rb1 on isoproterenol-induced acute myocardial ischemia in rats and its mechanism of action. *Chinese Journal of Experimental Traditional Medical Formulae*.

[22] Liu C. E., Wu S. X., Ye G. (2012). Mechanism of ginsenoside Rb1 against myocardial apoptosis during ischemia-reperfusion injury in diabetic rats. *Journal of Emergency in Traditional Chinese Medicine*.

[23] Liu F. Y. (2008). *Effects of Ginsenoside Rb1 on Blood Vessel Regeneration and Heart Function in Rats with Acute Myocardial Infarction*.

[24] Li P. (2006). *Effects of Ginsenoside Rb1 on Ventricular Remodeling in Rats with Acute Myocardial Infarction*.

[25] Zeng H. S., Liu Z. X., Liu X. C. (2003). Effect of ginsenoside-Rb1 and Re against cardiomyocyte apoptosis and expression of the related gene proteins in the experimental cardiac ischemia-reperfusion in rats. *Chinese Journal of Physical Medicine and Rehabilitation*.

[26] Wang Z., Li M., Wu W. K., Tan H. M., Geng D. F. (2008). Ginsenoside Rb1 preconditioning protects against myocardial infarction after regional ischemia and reperfusion by activation of phosphatidylinositol-3-kinase signal transduction. *Cardiovascular Drugs and Therapy*.

[27] Wu Y., Xia Z. Y., Dou J. (2011). Protective effect of ginsenoside Rb1 against myocardial ischemia/reperfusion injury in streptozotocin-induced diabetic rats. *Molecular Biology Reports*.

[28] Schmucker C., Bluemle A., Briel M. (2013). A protocol for a systematic review on the impact of unpublished studies and studies published in the gray literature in meta-analyses. *Systematic Reviews*.

[29] Blankstein R., Ahmed W., Bamberg F. (2012). Comparison of exercise treadmill testing with cardiac computed tomography angiography among patients presenting to the emergency room with chest pain: the rule out myocardial infarction using computer-assisted tomography (ROMICAT) study. *Circulation: Cardiovascular Imaging*.

[30] Menazza S., Sun J., Appachi S. (2017). Non-nuclear estrogen receptor alpha activation in endothelium reduces cardiac ischemia-reperfusion injury in mice. *Journal of Molecular and Cellular Cardiology*.

[31] Curbel P. A., MacCord C. S., Anderson R. D. (1994). A canine model of acute coronary artery thrombosis for the evaluation of reperfusion strategies. *Cardiology*.

[32] Kolk M. V. V., Meyberg D., Deuse T. (2009). LAD-ligation: a murine model of myocardial infarction. *Journal of Visualized Experiments*.

[33] Arteaga M. C., Ferro F. G., Villanueva S. O. (2002). 99mTc-glucarate for detection of isoproterenol-induced myocardial infarction in rats. *International Journal of Pharmaceutics*.

[34] Jin J. Y. (2008). Establishment an of animal model of myocardial infarction. *Chinese Journal of CT and MRI*.

[35] Chu J., Xu M. Q., He H., Chen K., Ye S., Zhai Z. (2002). Experimental study of acute myocardial infarction model in dogs. *Anhui Medical Journal*.

[36] Thorpe P. E., Hunter W. J., Zhan X. X., Dovgan P. S., Agrawal D. K. (1996). A non-injury diet-induced model of atherosclerosis for cardiovascular interventional research. *Angiology*.

[37] Rashid S. T., Salacinski H. J., Hamilton G., Seifalian A. M. (2004). The use of animal models in developing the discipline of cardiovascular tissue engineering: a review. *Biomaterials*.

[38] Baldwin H. S. (1999). Advances in understanding the molecular regulation of cardiac development. *Current Opinion in Pediatrics*.

[39] Turk J. R., Laughlin M. H. (2004). Physical activity and atherosclerosis: which animal model?. *Canadian Journal of Applied Physiology*.

[40] Moghadasian M. H., Frohlich J. J., McManus B. M. (2004). Advances in experimental dyslipidemia and atherosclerosis. *Laboratory Investigation*.

[41] Wolfensohn S., Lloyd M. (2000). Handbook of Laboratory Animal Management and Welfare. *Canadian Veterinary Journal*.

[42] Yellon D. M., Hausenloy D. J. (2007). Myocardial reperfusion injury. *The New England Journal of Medicine*.

[43] Downey J. M., Cohen M. V. (2009). Why do we still not have cardioprotective drugs?. *Circulation Journal*.

[44] Hausenloy D. J., Botker H. E., Condorelli G. (2013). Translating cardioprotection for patient benefit: position paper from the Working Group of Cellular Biology of the Heart of the European Society of Cardiology. *Cardiovascular Research*.

[45] Xu M. (2014). Research on main mechanisms of myocardial ischemia reperfusion injury and related drug therapy. *Practical Pharmacy And Clinical Remedies*.

[46] Wu Y., He L. (2010). Advances in research on mechanisms of myocardial ischemia-reperfusion injury and related therapeutic drugs. *Progress in Pharmaceutical Sciences*.

[47] Bodiga S., Wang W., Oudit G. Y. (2011). Use of ginseng to reduce post-myocardial adverse myocardial remodeling: applying scientific principles to the use of herbal therapies. *Journal of Molecular Medicine*.

[48] Hackam D. G., Redelmeier D. A. (2006). Translation of research evidence from animals to humans. *JAMA*.

[49] Moher D., Avey M., Antes G., Altman D. G. (2015). The National Institutes of Health and guidance for reporting preclinical research. *BMC Medicine*.

[50] Kilkenny C., Browne W. J., Cuthill I. C., Emerson M., Altman D. G. (2010). Improving bioscience research reporting: the ARRIVE guidelines for reporting animal research. *PLoS Biology*.

[51] Baker D., Lidster K., Sottomayor A., Amor S. (2014). Two years later: journals are not yet enforcing the ARRIVE guidelines on reporting standards for pre-clinical animal studies. *PLoS Biology*.

[52] Hackam D. G. (2007). Translating animal research into clinical benefit. *British Medical Journal*.

[53] Rongen G. A., Wever K. E. (2015). Cardiovascular pharmacotherapy: innovation stuck in translation. *European Journal of Pharmacology*.

[54] Glasziou P., Vandenbroucke J. P., Chalmers I. (2004). Assessing the quality of research. *British Medical Journal*.

